# Early Diagnosis of Relapsing Polychondritis With Airway Involvement: A Case Report

**DOI:** 10.7759/cureus.81101

**Published:** 2025-03-24

**Authors:** Natsumi Kushima, Toyoshi Yanagihara, Naoko Himuro, Yuki Shundo, Naoki Hamada, Masaki Fujita

**Affiliations:** 1 Department of Respiratory Medicine, Fukuoka University Hospital, Fukuoka, JPN; 2 Department of Nephrology and Rheumatology, Fukuoka University Hospital, Fukuoka, JPN

**Keywords:** airway chondritis, bronchoscopy, relapsing polychondritis, tracheal stenosis, tracheomalacia

## Abstract

Relapsing polychondritis (RP) is a rare systemic immune-mediated disease that causes recurrent inflammation of cartilaginous tissues. Airway involvement is a significant prognostic factor; however, diagnosis is often delayed because of nonspecific symptoms. We report the case of a 38-year-old woman who presented with pharyngeal discomfort, cough, and chest pain. Initial tests revealed elevated inflammatory markers and anemia. A chest CT scan showed soft tissue enhancement around the costal cartilage and thickening of the tracheal and bronchial walls. Bronchoscopy demonstrated extensive inflammation from the larynx to the main bronchi, with approximately 90% narrowing of the left bronchus during exhalation, suggestive of tracheomalacia. Based on clinical findings, imaging, bronchoscopy, and serological tests, a diagnosis of RP was made, fulfilling McAdam's and Damiani's criteria. Although pulmonary function tests continued to show obstructive ventilatory impairment, treatment withcorticosteroids (0.6 mg/kg prednisolone) and methotrexate resulted in rapid improvement in inflammatory markers and imaging findings. This case highlights the importance of early bronchoscopy in assessing airway involvement in RP, even when respiratory symptoms are mild, and emphasizes the need to balance the diagnostic benefits against potential risks, particularly in those with reduced forced vital capacity. Early diagnosis and timely intervention, including consideration of biologics, may prevent progression to severe airway compromise.

## Introduction

Relapsing polychondritis (RP) is a rare systemic immune-mediated disease characterized by chronic and recurrent inflammation of cartilage in various organs, including auricular, tracheobronchial, nasal, and articular regions [1]. Airway involvement is an important prognostic factor; however, its nonspecific symptoms and low incidence often result in delayed diagnosis [2]. Currently, there are no standard guidelines for the evaluation and treatment of airway lesions in RP. Recent studies have highlighted the importance of dynamic chest CT, particularly expiratory imaging, in diagnosing airway involvement [3]. Expiratory CT can detect airway collapse in up to 94% of RP patients, compared to 47% with standard inspiratory CT [4]. However, conventional chest CT also plays a crucial role in identifying airway abnormalities such as tracheal thickening and stenosis. Additionally, bronchoscopy allows for dynamic assessment of airway lesions in real time, providing valuable insights into airway stability, inflammation, and structural abnormalities. Recognizing the diverse clinical phenotypes of RP, especially those with predominant respiratory involvement, is crucial for predicting disease progression and optimizing treatment strategies [3,5]. Here, we present a case of RP with airway chondritis, which was diagnosed relatively early using conventional chest CT and bronchoscopy.

## Case presentation

A 38-year-old woman was referred to our department for evaluation of possible airway lesions. Four months prior, she experienced discomfort in her pharyngeal area. She visited an otolaryngologist and was diagnosed with an upper respiratory tract infection. She was treated with tranexamic acid, ambroxol, montelukast, and dextromethorphan; however, her symptoms persisted. During that visit, anemia was noted, prompting both upper and lower gastrointestinal endoscopic examinations, which were unremarkable. Three months prior, due to a persistent cough, the patient sought further evaluation. A chest X-ray was normal, but blood tests revealed a mild elevation in C-reactive protein (CRP). Two months before presentation, she developed stiffness in her back and anterior chest pain­, which was most severe upon waking and exacerbated by deep breathing or coughing. One month prior, an elevated platelet count and increased CRP led to her referral to our hospital for further assessment. A chest CT scan revealed enhanced soft tissue shadows around the costal cartilage, blurring of the adipose tissue, and thickening of the trachea and main bronchi walls, raising suspicion of RP.

**Figure 1:**
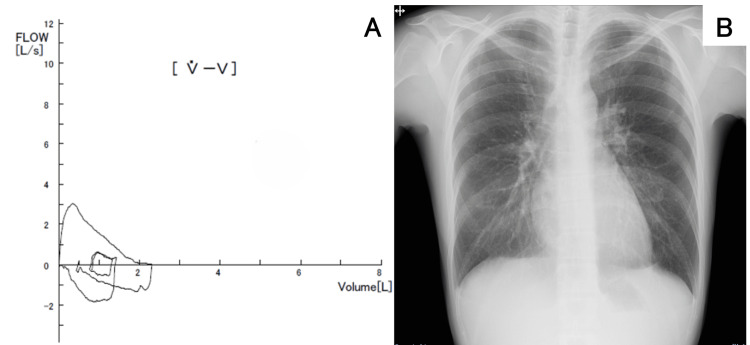
(A) Flow volume curve. (B) Chest X-ray (A) Pulmonary function tests revealed obstructive ventilatory impairment with FVC 2.50L (83.9%) and FEV1 1.70L (66%). (B) The chest X-ray revealed no significant abnormalities. FVC: forced vital capacity; FEV1: forced expiratory volume in one second

At the initial examination, her vital signs were as follows: pulse rate: 82/min, blood pressure: 117/72 mmHg, body temperature: 36.9°C, respiratory rate: 16/min, and SpO2: 98% (room air). There was no redness or swelling of the ear lobes or the nasal cartilage, although the right conjunctiva was congested. Chest auscultation was unremarkable. There was no tenderness or swelling in the joints. No obvious skin rashes were noted. Despite minimal respiratory symptoms and no reported shortness of breath, pulmonary function tests revealed obstructive ventilatory impairment with forced vital capacity (FVC) of 2.50 L (83.9% predicted) and forced expiratory volume in one second (FEV1) of 1.70 L (66% predicted) (Figure 1A). Laboratory studies revealed nonspecific inflammatory markers, including elevated erythrocyte sedimentation rate (ESR) and CRP. The serum matrix metalloproteinase-3 (MMP-3) level was high, suggesting articular cartilage destruction. Anti-type II collagen antibody was positive, while antibodies associated with collagen diseases were negative (Table 1). The chest radiographs were unremarkable (Figure 1B). 

**Table 1:** Labolatory results before treatment ESR: erythrocyte sedimentation rate; IgG: immunoglobulin G; IgA: immunoglobulin A; IgM: immunoglobulin M; CRP: C-reactive protein; MMP-3: matrix metalloproteinase-3; RF: rheumatoid factor; CCP: cyclic citrullinated peptide; PR3-ANCA: proteinase-3-antineutrophil cytoplasmic antibody; MPO-ANCA: myeloperoxidase-antineutrophil cytoplasmic antibody; Agal-IgG: Anti galactose-deficient immunoglobulin G; ANA: antinuclear antibody; sIL-2R: soluble interleukin-2 receptor

Test	Values	Unit	Reference range
Hematology			
WBC	5.6	×10^3/μL	3.3-8.6
Neutrophils	68	%	38-74
Eosinophils	1.5	%	0-8.5
Lymphocytes	25.5	%	16.5-49.5
Monocytes	4.5	%	2.0-10.0
Hb	11.3	g/dL	11.6-14.8
Plt	556	×10^3/μL	158-348
ESR	>=120	mm/h	3.0-15.0
Biochemistry			
TP	8.3	g/dL	6.6-8.1
Alb	3.3	g/dL	4.1-5.1
AST	14	U/L	13-30
ALT	15	U/L	7.0-23
LDH	113	U/L	124-222
BUN	6	mg/dL	8.0-20
Cr	0.49	mg/dL	0.46-0.79
CK	25	U/L	41-153
Ferritin	336	ng/mL	6.23-138
IgG	2215	mg/dL	861-1747
IgA	322	mg/dL	93-393
IgM	86	mg/dL	50-269
Serology			
CRP	8.17	mg/dL	0-0.14
MMP-3	77.5	ng/mL	17.3-59.7
RF	10	U/mL	<=15
Anti-CCP Ab	<0.6	U/mL	<4.5
Agal-IgG	2.3	AU/mL	<6.0
ANA(FANA)	<40	times	<40
Anti dsDNA Ab	<10	U/mL	<10
PR3-ANCA	1.4	U/mL	<3.5
MPO-ANCA	<1.0	U/mL	<3.5
Anti-type II collagen Ab	73.6	EU/mL	<20
sIL-2R	461	U/mL	121-613
Protein fraction			
Alb	43.4	%	55.8-66.1
α1	7	%	2.9-4.9
α2	14.3	%	7.1-11.8
β1	6.3	%	4.7-7.2
β2	6.7	%	3.2-6.5
γ	1.3	%	11.1-18.8
Urinalysis			
Protein	(-)		
Blood	(-)		

**Figure 2:**
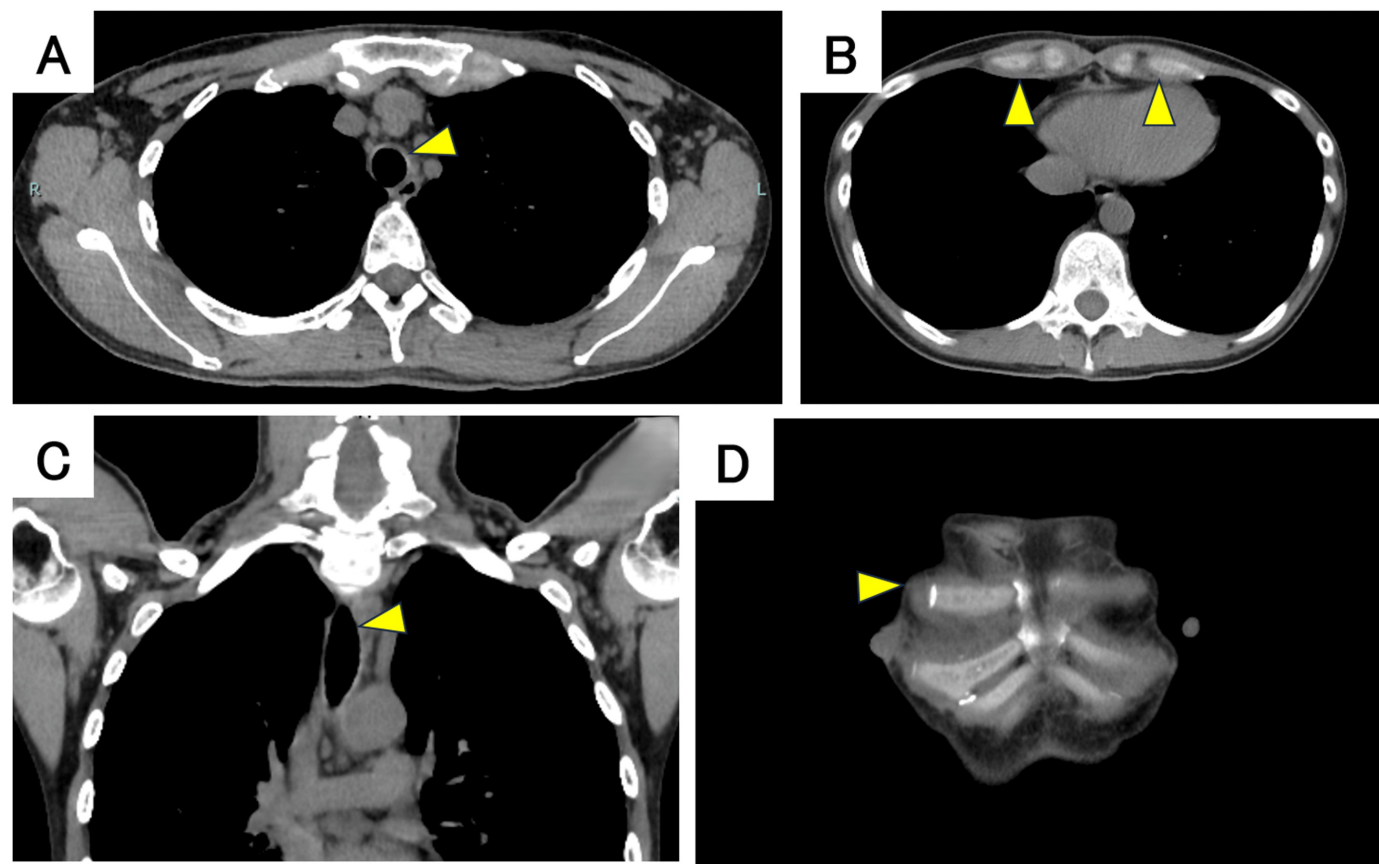
Chest CT scan of the patient. (A, C) The arrows indicate the tracheal wall thickening. (B, D) The arrows indicate the increased soft tissue shadow around the costal cartilage and unclear fatty tissue.

**Figure 3:**
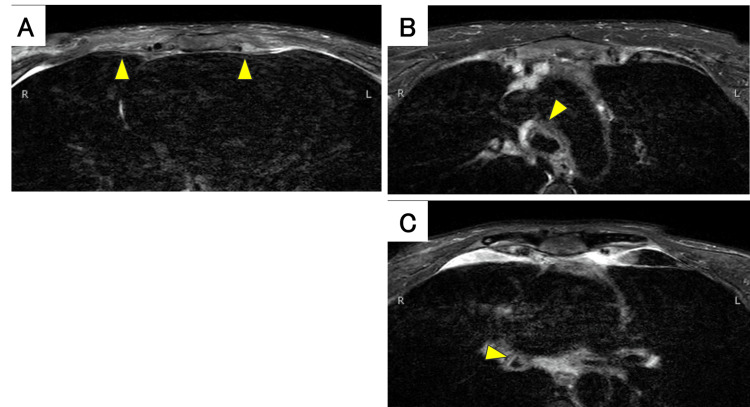
The MRI of the chest (A) High signal with unclear boundaries was observed in both parasternal regions, the anterior wall, and the intercostal region on the fat-suppressed T2-weighted images. (B, C) The thickening of the trachea and bronchial wall was also confirmed.

**Figure 4:**
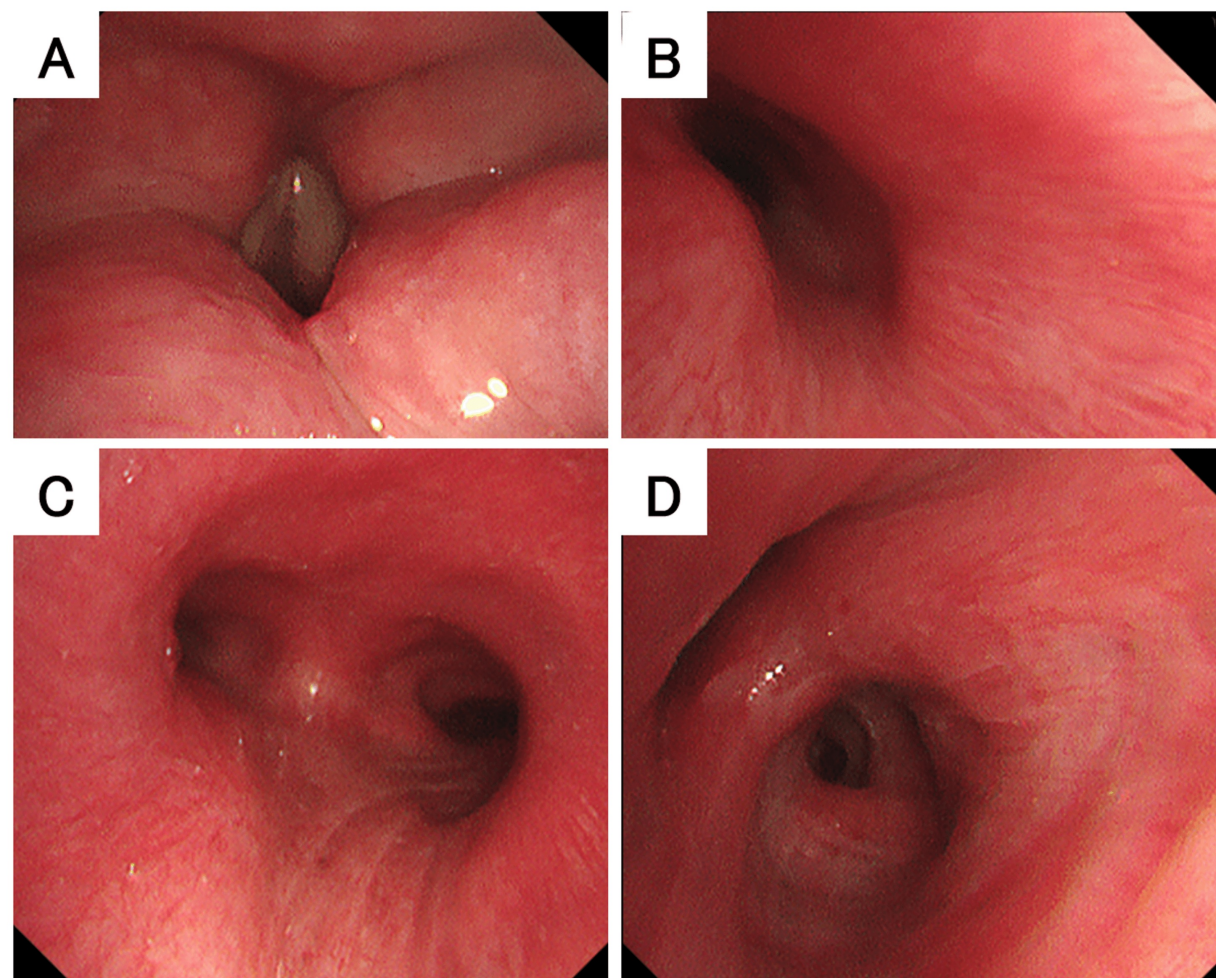
The bronchoscopy findings. (A) In the larynx, swelling of the arytenoid cartilage was noticeable. (B-D) The tracheobronchi were red and swollen all around. (B, D) The left bronchus collapsed during expiration.

The chest CT scan showed thickening of the tracheal and main bronchial walls and increased soft tissue shadows with indistinct fat planes around the costal cartilage (Figure 2). Chest MRI revealed high signal intensity with unclear margins in both parasternal regions, the anterior chest wall, and the intercostal spaces on fat-suppressed T2-weighted images; tracheal and bronchial wall thickening was also confirmed (Figure 3). Bronchoscopy was performed after obtaining informed consent from the patient. Bronchoscopy showed swelling of the arytenoid cartilage and the circumferential redness and edema of the tracheal and main bronchial epithelium (Figure 4). Notably, the left bronchus exhibited approximately 90% luminal narrowing during exhalation. While the patient presented with respiratory symptoms, the presence of joint pain and other inflammatory findings led to the exclusion of asthma. Vasculitis was excluded due to the absence of peripheral neuropathy, skin manifestations, renal involvement, and negative autoantibodies. Based on the presence of non-erosive, seronegative, inflammatory polyarthritis, ocular inflammation, and airway chondritis, the patient met McAdam's criteria [1] and modified Damiani criteria [6], leading to a diagnosis of RP.

**Figure 5:**
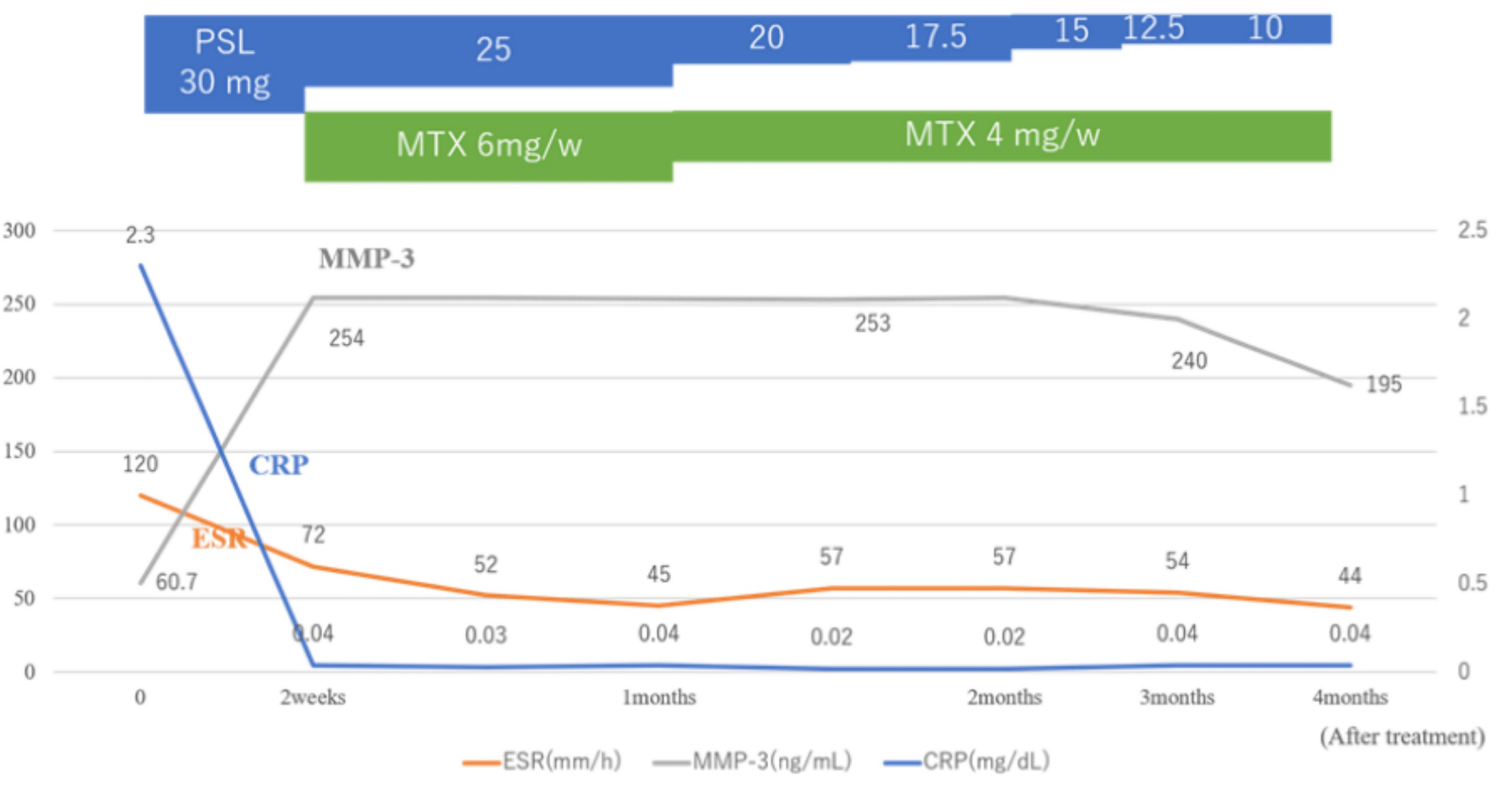
Clinical course of the patient. The CRP rapidly decreased and the chest pain was reduced after starting treatment with prednisolone at 30 mg/day (0.6 mg/kg). Subsequently, methotrexate was added and the steroid was gradually tapered.

**Figure 6:**
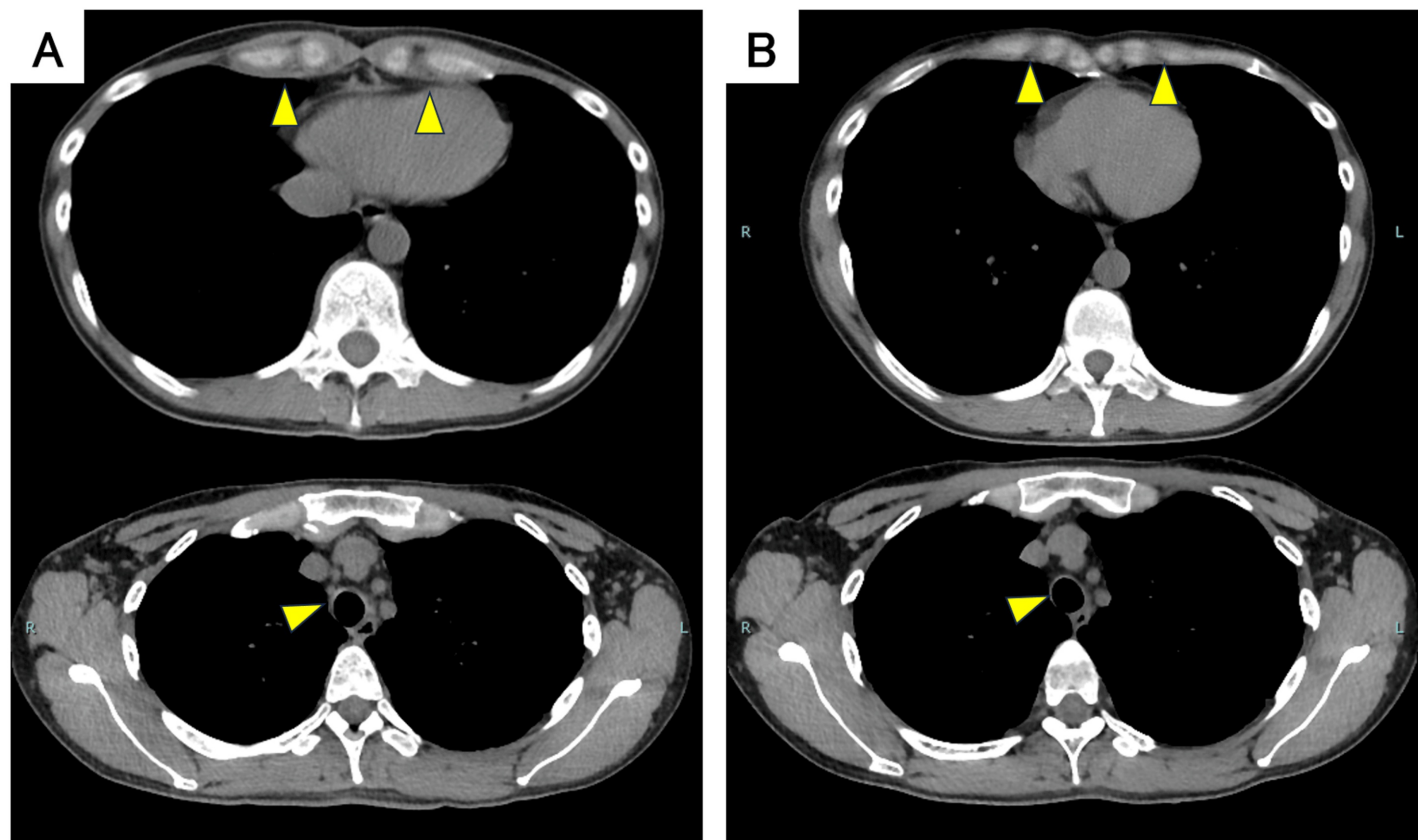
Comparison of chest CT findings (A) before and (B) two months after treatment. These showed a reduction in the soft tissue shadow thickening around the costal cartilage and in the thickening of the tracheal and bronchial walls.

Following the initiation of treatment with prednisolone at 0.6 mg/kg orally, CRP levels rapidly decreased, and the chest pain improved. Methotrexate (MTX) at 6 mg/week was subsequently added but was later reduced to 4 mg/week, and the steroid was gradually tapered (Figure 5). Two months after treatment initiation, a follow-up CT scan showed reduced soft tissue thickening around the costal cartilage and decreased thickening of the tracheal and bronchial walls (Figure 6). However, the patient later developed hoarseness and difficulty breathing while supine. Pulmonary function tests at two- and three months post-treatment continued to show residual obstructive ventilatory impairment. With improvement in inflammatory markers and stabilization of respiratory symptoms, combined therapy with steroids and MTX is ongoing.

## Discussion

Laryngotracheobronchial involvement is present at initial presentation in only about 10% of RP cases, but it eventually develops in nearly 50% of patients, more commonly in females [7]. Airway lesions can affect the trachea, bronchi, and larynx, and symptoms vary depending on the location and extent of involvement, ranging from cough, sputum, dyspnea, wheezing, hoarseness, and dysphagia. Historically, the time from symptom onset to diagnosis has averaged 2.9 years [2]. When respiratory symptoms are the only findings, RP is often misdiagnosed (for example, asthma) [8]. In cases where typical signs such as auricular chondritis are absent, as in our patient, the diagnosis of RP is at high risk of being delayed. Because airway involvement can lead to life-threatening stenosis and obstruction, early treatment is critical. However, given the rarity of RP, there are no established evaluation methods or treatment guidelines.

In this case, diagnosis was achieved approximately four months after symptom onset. Although the chief complaint was chest pain, with only mild cough and pharyngeal discomfort, and CT findings were subtle, bronchoscopy revealed more extensive inflammation, particularly in the left bronchus where tracheomalacia was evident. Tracheomalacia in the adult population is typically due to an acquired injury from previous surgery involving the airway, intubation, or chronic lung disease. Additionally, chronic compression can occur due to goiter or tumors and masses. Inflammatory processes such as RP can also weaken the structure of the airway, predisposing it to collapse [9,10].

Bronchoscopic examination is crucial in confirming airway involvement in RP, particularly when symptoms are mild or nonspecific [11,12]. Fujita et al. also reported that bronchoscopy was useful in the long-term observation of airway lesions due to RP [13]. Typical bronchoscopic findings include: (1) Mucosal erythema and edema, as seen in our case, indicating early inflammation [14]; (2) progressive subglottic and tracheal stenosis due to fibrosis [15]; (3) tracheobronchomalacia from cartilage destruction, resulting in dynamic airway collapse during exhalation [15].

Despite its diagnostic benefits, bronchoscopy carries risks in RP patients--especially in those with reduced FVC, who are at higher risk of complications, such as bronchospasm, airway edema, and respiratory failure [16]. Therefore, the decision to perform bronchoscopy should be individualized. In our case, the procedure was carried out in a controlled setting with emergency airway management preparations. This case reinforces the importance of balancing diagnostic benefits and procedural risks when evaluating airway involvement in RP.

Non-invasive imaging, particularly dynamic expiratory CT, is recommended as a first-line tool for assessing tracheobronchomalacia [17]. Studies indicate that expiratory CT can detect airway collapse in up to 94% of RP patients, compared to 47% with standard inspiratory CT [4]. Although useful for follow-up, CT may be inconclusive or insufficient when tissue sampling is required, making bronchoscopy indispensable.

This case demonstrates that progressive airway involvement may occur even when systemic symptoms and inflammatory markers improve. Zhai et al. described a cohort of RP patients with airway involvement where progressive airway stenosis and tracheomalacia were significant concerns, often requiring interventions like tracheostomy or stenting [5]. However, they carry significant risks. Currently, treatment for RP, primarily systemic corticosteroids and immunosuppressants, is largely empirical [7]. While systemic corticosteroids remain the cornerstone of initial management, their long-term use is associated with significant morbidity, prompting exploration of alternative and adjunctive therapies. The role of steroid-sparing agents like methotrexate and azathioprine is debated, with varying efficacy reported in different cohorts [11]. More recently, biologics, particularly anti-TNFα antibodies, have shown promise in managing RP, especially in reducing inflammatory burden and potentially preventing structural damage. Handa et al. reported significantly higher survival rates in RP patients with airway involvement who received biologics, suggesting that early use may prevent the need for stenting and improve long-term outcomes [14]. This aligns with the observation in our case. While our patient's systemic symptoms improved initially, the insidious progression of airway disease emphasizes the need for vigilant monitoring, even in the face of seemingly controlled systemic inflammation. Given the patient’s young age, careful long-term management of airway lesions is essential. The potential for early intervention with biologics, as suggested by Handa et al., needs to be balanced against the cost and accessibility issues, highlighting the ongoing need for research into effective and affordable treatment strategies for RP-related airway disease [14].

## Conclusions

Even when respiratory symptoms are mild, significant airway inflammation can be present in RP. In our case, bronchoscopy revealed marked epithelial redness and swelling, and dynamic bronchial narrowing-findings that indicated tracheomalacia. Since airway lesions have a major impact on prognosis in RP, safe and thorough evaluation of airway involvement is critical. Standardized guidelines are needed to optimize management. Early, appropriate treatment may help prevent severe complications.
